# All-epiphyseal anterior cruciate ligament reconstruction yields superior sports performances than the trans-epiphyseal technique in skeletally immature patients: a systematic review

**DOI:** 10.1186/s10195-024-00751-9

**Published:** 2024-02-20

**Authors:** Filippo Migliorini, Marco Pilone, Michael Kurt Memminger, Jörg Eschweiler, Riccardo Giorgino, Nicola Maffulli

**Affiliations:** 1 Department of Orthopaedic, Trauma, and Reconstructive Surgery, RWTH University Hospital, Pauwelsstraße 30, 52074 Aachen, Germany; 2 Department of Orthopaedics and Trauma Surgery, Academic Hospital of Bolzano (SABES-ASDAA), 39100 Bolzano, Italy; 3 Residency Program in Orthopedics and Traumatology, University of Milan, Milan, Italy; 4 Department of Trauma and Reconstructive Surgery, BG Hospital Bergmannstrost, Halle, Germany; 5 Department of Medicine and Psychology, University of Rome “La Sapienza”, Rome, Italy; 6 School of Pharmacy and Bioengineering, Faculty of Medicine, Keele University, ST4 7QB Stoke on Trent, England; 7 Barts and the London School of Medicine and Dentistry, Centre for Sports and Exercise Medicine, Queen Mary University of London, Mile End Hospital, E1 4DG London, England

**Keywords:** ACL, Anterior cruciate ligament, All-epiphyseal, Trans-epiphyseal, Skeletally immature patients, Open physis

## Abstract

**Background:**

Anterior cruciate ligament (ACL) tears in skeletally immature patients are increasingly common. Evidence comparing the outcomes of all-epiphyseal versus trans-epiphyseal ACL reconstruction in skeletally immature patients is limited, and the current literature could benefit from a comprehensive systematic review. The present study compared all-epiphyseal versus trans-epiphyseal ACL reconstruction in skeletally immature patients. The outcomes of interest were to compare joint laxity, patient-reported outcome measures (PROMs), return to sport, and complications.

**Methods:**

This study was conducted according to the 2020 Preferred Reporting Items for Systematic Reviews and Meta-Analyses (PRISMA) statement. In November 2023, the following databases were accessed: PubMed, Web of Science, Google Scholar, and Embase. No additional filters were used in the database search. All the clinical studies investigating ACL reconstruction in skeletally immature patients were accessed. Only articles that clearly stated the surgical technique (all- or trans-epiphyseal) were eligible. Only articles with a minimum of 6 months of follow-up were included. Only articles that clearly stated that surgeries were conducted in children with open physis were eligible.

**Results:**

Data from 1489 patients (1493 procedures) were collected, of which 32% (490 of 1489 patients) were female. The mean length of follow-up was 46.6 months. The mean age of the patients was 12.7 years. No difference was found in joint laxity (Table 3): positive pivot shift (*P* = 0.4), positive Lachman test (*P* = 0.3), and mean arthrometer laxity (*P* = 0.1). No difference was found in PROMs (Table 4): International Knee Documentation Committee (IKDC) (*P* = 0.3), Lysholm (*P* = 0.4), and Tegner (*P* = 0.7). The trans-epiphyseal technique was associated with a greater rate of patients unable to return to sports (1% versus 7%, *P* = 0.0001) and with a longer time to return to sports (7.7 versus 8.6 months, *P* = 0.01). Though the trans-epiphyseal technique was associated with a lower rate of return to sport, this difference was not statistically significant (*P* = 0.8). No difference was evidenced in the rate of patients who had reduced their league or level of sports activity (*P* = 0.6) or in the rate of patients who had returned to their previous league or level of sports activity (*P *= 0.7). No difference was found in the rate of complication: re-tear (*P* = 0.8), reoperation (*P* = 0.7), increased laxity (*P* = 0.9), and persistent instability sensation (*P* = 0.3).

**Conclusion:**

Trans-epiphyseal ACL reconstruction was associated with a greater rate of patients unable to return to sport and with a longer time to return to sport compared with the all-epiphyseal technique in skeletally immature patients.

*Level of evidence* Level III, systematic review.

## Introduction

An anterior cruciate ligament (ACL) tear in skeletally immature patients is increasingly common [1, 2], with an estimated incidence worldwide of 70 per 100,000 injuries per year [3–7]. The prevalence of ACL tears in children with open physis has increased over the last 20 years [8–12]. ACL injury in the young athletic population occurs during jumping, twisting, and cutting movements [13]. ACL deficiency affects the knee biomechanics, increasing the anteroposterior translation of the femur over the tibia [14–16]. Laxity may result in joint instability sensation, articular cartilage injuries, and meniscal damage [15–28]. The optimal management of ACL, conservative rather than surgical, is still debated [29, 30].

ACL reconstruction in skeletally immature patients aims to restore knee stability, preventing further soft tissue injuries and preserving physiological growth of the lower limb [31–33]. Surgery in the pediatric population is debated [34–36]. Damaging the epiphyseal plates could lead to growth disturbances, including leg-length discrepancy or an angular deformity [9, 37–39]. In children with open physis, both all-epiphyseal and trans-epiphyseal ACL reconstruction have been described. The trans-epiphyseal technique is similar to the procedure performed in adults and consists of a femoral and tibia tunnel, where the graft is allocated and fixed [40–42]. The all-epiphyseal technique restores the anatomic ACL footprint with unique tunnel drilling and fixation techniques. Several all-epiphyseal ACL reconstruction techniques have been described [31, 43–45]; in these techniques the femoral and tibial tunnels are drilled entirely within the physis, leaving the growth plates untouched [46]. Evidence comparing the outcomes of all-epiphyseal versus trans-epiphyseal ACL reconstruction in skeletally immature patients is limited, and to the best of our knowledge, the current literature could benefit from a comprehensive systematic review.

The present study compared all-epiphyseal versus trans-epiphyseal ACL reconstruction in skeletally immature patients. The outcomes of interest were to compare joint laxity, PROMs, return to sport, and complications.

## Methods

### Eligibility criteria

All the clinical studies investigating ACL reconstruction in skeletally immature patients were accessed. Only studies published in peer-reviewed journals were considered. According to the author language capabilities, articles in English, German, Italian, French, and Spanish were eligible. Only studies with levels I–III of evidence, according to the Oxford Centre of Evidence-Based Medicine [47], were considered. Reviews, opinions, letters, and editorials were not considered. Animals, in vitro, biomechanics, computational, and cadaveric studies were not eligible. Only articles that clearly stated the surgical technique (all- or trans-epiphyseal) were eligible. Only articles with a minimum of 6 months of follow-up were included. Only articles that clearly stated that surgeries were conducted in children with open physis were eligible. Missing quantitative data under the outcomes of interests warranted the exclusion of the study.

### Search strategy

This study was conducted according to the Preferred Reporting Items for Systematic Reviews and Meta-Analyses: the 2020 PRISMA statement [48]. The Problem, Intervention, Comparison, Outcomes, Timing (PICOT) algorithm was preliminarily established:

- P (Problem): ACL tears;
- I (Intervention): all-epiphyseal ACL reconstruction;
- C (Comparison): trans-epiphyseal ACL reconstruction;
- O (Outcomes): laxity, PROMs, return to sport, complications;
- T (Timing): minimum 6-month follow-up.

In November 2023, the following databases were accessed: PubMed, Web of Science, Google Scholar, and Embase. No time constraint was set for the search. The medical subject headings used for the database search are described in the appendix. No additional filters were used in the database search.

### Selection and data collection

Two authors (R.G. and J.E) independently performed the database search. All the resulting titles were screened by hand and, if suitable, the abstract was accessed. The full texts of the abstracts that matched the topic of interest were accessed. If the full text was not accessible or available, the article was not considered for inclusion. A cross reference of the bibliography of the full-text articles was also performed for inclusion. Disagreements were debated and mutually solved by the authors. In case of further disagreements, a third senior author (N. M.) made the final decision.

### Data items

Two authors (R.G. and J.E.) independently performed data extraction. The following data at baseline were extracted: author, year of publication and journal, length of follow-up, male:female ratio, number of patients with related mean age and body mass index (BMI). To investigate knee stability, data from the manual (pivot shift and Lachman tests) and instrumental laxity were extracted. Instrumental laxity was typically evaluated using the arthrometers KT-1000 and KT-2000 (MEDmetric Corp, San Diego, California). Both of these devices applied a force of 134N on the tibial plateau over the femoral condyles, directed anteriorly. Data concerning the following PROMs were collected at baseline and at the last follow-up: Tegner Activity Scale [49], Lysholm Knee Scoring Scale [50], and IKDC [51]. The minimum clinically important difference (MCID) for the Lysholm score was 10/100, 15/100 for the IKDC, and 0.5/10 for the Tegner score [52–54]. To evaluate return to sport, the following data were extracted: mean return to sport, rate of patients unable to return to sport, rate of return to sport, rate of patients who had reduced their league or level of sports activity, and rate of patients who had returned to their previous league or level of sports activity. Data on the following rates of complication were collected: re-tear, re-operation, increased laxity, and persistent instability sensation. Re-tear was defined as a further postoperative tear of the ACL documented at imaging. Any surgical revision following failure of the indexed ACL reconstruction was considered as a re-operation. Data were extracted in Microsoft Office Excel version 16.72 (Microsoft Corporation, Redmond, USA).

### Assessment of the risk of bias

The methodological quality of the included studies was assessed by two authors independently (R.G. and J.E.) using the Coleman Methodology Score (CMS) [55]. Disagreements were discussed and resolved by consensus. In addition, Coleman criteria also assess the quality of outcome reports. In detail, the following criteria are included for the assessment: population size, length of follow-up, surgical approach used, study design, description of diagnosis, surgical technique, and rehabilitation, as well as outcome criteria assessment and the subject selection process. Subscores for each domain were added for a total possible score of 100. The quality of the studies is scored between 0 (poor) and 100 (excellent). A mean value greater than 60 points was considered satisfactory.

### Synthesis methods

The statistical analyses were performed by the main author (F.M.) following the recommendations of the Cochrane Handbook for Systematic Reviews of Interventions [56]. The software IBM SPSS version 25 was used. For descriptive statistics, mean and standard deviation or the observed frequency (number of cases divided by the number of included patients) were used. The mean difference (MD) effect measure was calculated to compare continuous outcomes and the odds ratio (OR) for binary data. The confidence interval (CI) was set at 95%. The *t*-test and $\chi$^2^ tests were performed for continuous and binary variables, respectively, with a value of *P* < 0.05 considered statistically significant.

## Results

### Study selection

**Fig. 1:**
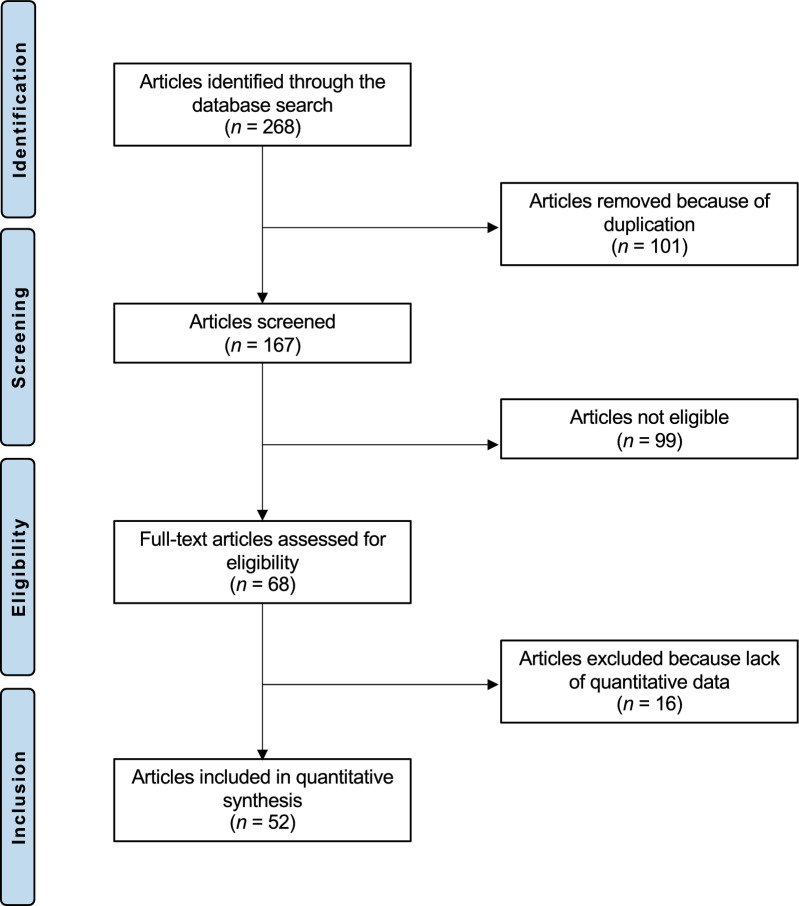
PRISMA flow chart of the literature search

A total of 268 articles were identified through the systematic literature search. After the assessment of titles and abstracts, 101 studies were identified as duplicates and excluded. Insufficient fulfillment of the eligibility criteria led to the exclusion of 99 additional studies. Reasons for exclusion were: inappropriate study design (*N* = 46), lack of clarity that treatment was provided only to patients with open physis (*N* = 12), not clearly stating the surgical technique (*N* = 10), low level of evidence (*N* = 7), language limitations (*N* = 14), follow-up shorter than 6 months (*N* = 10). An additional 16 studies were excluded after full-text review as they did not include quantitative data on outcomes of interest. This left 52 studies to be included in the quantitative synthesis. Of them, two were prospective and 50 were retrospective studies. A trans-epiphyseal reconstruction technique was used in 29 studies, all-epiphyseal reconstruction in 22 studies, and one trial reported data from both procedures. The results of the literature search process are shown in Fig. 1.

### Methodological quality assessment

According to the CMS, the follow-up time was acceptable in all articles reviewed. The number of patients enrolled exceeded 10 patients in 94.2% (49 of 52) of the studies assessed. Limitations identified by the CMS score included the retrospective study design in 96.2% (50 of 52) of the included studies. Confounding was frequently found with outcome measures and the assessment process. The poor quality of surgical protocols and of the diagnoses descriptions, and the lack of standardized postoperative rehabilitation programs in most studies resulted in fair reliability. Concluding, the CMS resulted in 61.4 ± 6.9 points, attesting to the fair quality of the methodology of the investigations included in the present study (Table 1).

**Table 1:** Generalities and patient baseline of the included studies

Author and year	Journal name	Design	CMS	Follow-up (*months*)	Technique	Patients (*n*)	Knees (*n*)	Mean age	Female (*n*)
Aichroth et al. [57]	*J Bone Joint Surg Br*	Prospective	77	49.0	Trans-epiphyseal	45	45	13.0	13
Akinleye et al. [58]	*Int J Sports Phys Ther*	Retrospective	48	36.0	All-epiphyseal	1	2	10.0	1
Andrews et al. [59]	*Am J Sports Med*	Retrospective	60	58.0	Trans-epiphyseal	8	8	13.0	0
Arbes et al. [60]	*Int Orthop*	Retrospective	54	64.8	Trans-epiphyseal	4	4	13.9	13
Aronowitz et al. [61]	*Am J Sports Med*	Retrospective	68	25.0	Trans-epiphyseal	19	19	13.4	10
Asai et al. [62]	*Sci Rep*	Retrospective	66	23.0	Trans-epiphyseal	27	27	13.9	16
Bonnard et al. [63]	*J Bone Joint Surg Br*	Retrospective	72	66.0	All-epiphyseal	56	56	12.2	13
Calvo et al. [64]	*Am J Sports Med*	Retrospective	64	127.2	Trans-epiphyseal	27	27	13.0	11
Cassard et al. [65]	*J Pediatr Orthop*	Retrospective	66	33.6	All-epiphyseal	28	28	13.0	8
Cohen et al. [66]	*Arthroscopy*	Retrospective	63	45.0	Trans-epiphyseal	26	26	13.3	15
Courvoisier et al. [67]	*Knee Surg Sports Traumatol Arthrosc*	Retrospective	69	36.0	Trans-epiphyseal	37	37	14.0	20
Cordasco et al. [68]	*Am J Sports Med*	Retrospective	60	32.1	All-epiphyseal	23	23	12.2	6
Cruz et al. [69]	*J Pediatr Orthop*	Retrospective	56	21.0	All-epiphyseal	103	103	12.1	24
Demange et al. [70]	*Am J Sports Med*	Prospective	58	219.6	Trans-epiphyseal	12	12	10.7	5
Foissey et al. [71]	*Arthrosc Sports Med Rehabil*	Retrospective	62	57.0	Trans-epiphyseal	20	20	13.6	6
				57.0	Trans-epiphyseal	20	20	14.0	2
Fuchs et al. [72]	*Arthroscopy*	Retrospective	62	40.0	Trans-epiphyseal	10	10	13.2	4
Gebhard et al. [73]	*Knee Surg Sports Traumatol Arthrosc*	Retrospective	63	32.0	Trans-epiphyseal	68	68	11.9	19
				33.0	Trans-epiphyseal	40	40	15.3	14
Goddard et al. [74]	*Am J Sports Med*	Retrospective	55	24.0	Trans-epiphyseal	32	32	13.0	11
Greenberg et al. [75]	*Sports Health*	Retrospective	69	15.4	All-epiphyseal	16	16	12.3	
Guzzanti et al. [76]	*Am J Sports Med*	Retrospective	52	69.2	All-epiphyseal	8	8	11.4	0
Hoshikawa et al. [77]	*Orthop J Sports Med*	Retrospective	55	52.7	All-epiphyseal	3	3	13.0	1
Hui et al. [78]	*Am J Sports Med*	Retrospective	54	25.0	Trans-epiphyseal	16	16	12.0	4
Koch et al. [79]	*Knee Surg Sports Traumatol Arthrosc*	Retrospective	60	54.0	All-epiphyseal	12	13	12.1	2
Kocher et al. [80]	*J Bone Joint Surg Am*	Retrospective	61	63.6	All-epiphyseal	44	44	10.3	
Kohl et al. [81]	*Knee*	Retrospective	58	49.2	Trans-epiphyseal	15	15	12.8	3
Kumar et al. [82]	*J Bone Joint Surg Am*	Retrospective	55	72.3	Trans-epiphyseal	32	32	11.3	4
Lanzetti et al. [83]	*Int Orthop*	Retrospective	62	96.1	All-epiphyseal	42	42	12.5	12
Lawrence et al. [44]	*Clin Orthop Relat Res*	Retrospective	48	12,0	All-epiphyseal	3	3	11,3	0
Lemaitre et al. [84]	*Orthop Traumatol Surg Res*	Retrospective	50	15.0	Trans-epiphyseal	13	14	13.6	
Liddle et al. [85]	*J Bone Joint Surg Br*	Retrospective	52	44.0	Trans-epiphyseal	17	17	12.1	3
Mauch et al. [86]	*Sports Med Arthrosc Rehabil Ther Technol*	Retrospective	50	> 60	Trans-epiphyseal	49	49	13.0	21
McCarroll et al. [87]	*Am J Sports Med*	Retrospective	72	26.4	Trans-epiphyseal	24	24	13.3	12
McCarroll et al. [88]	*Am J Sports Med*	Retrospective	66	50.4	Trans-epiphyseal	60	60	14.2	31
Mcintosh et al. [89]	*Arthroscopy*	Retrospective	59	41.1	Trans-epiphyseal	16	16	13.6	5
Micheli et al. [90]	*Clin Orthop Relat Res*	Retrospective	63	66.5	All-epiphyseal	8	8	11.0	1
Nakhostine et al. [91]	*J Pediatr Orthop*	Retrospective	50	52.8	All-epiphyseal	5	5	14.0	0
Nikolaou et al. [92]	*Knee Surg Sports Traumatol Arthrosc*	Retrospective	67	38.0	Trans-epiphyseal	94	94	13.7	38
Perelli et al. [93]	*Am J Sports Med*	Retrospective	70	26.6	All-epiphyseal	34	34	13.5	11
				25.1	All-epiphyseal	32	32	13.8	12
Pennock et al. [94]	*Orthop J Sports Med*	Retrospective	68	38.4	All-epiphyseal	26	26	11.8	?
Redler et al. [95]	*Arthroscopy*	Retrospective	62	43.4	Trans-epiphyseal	18	18	14,2	6
Robert et al. [96]	*Arthroscopy*	Retrospective	58	42.0	All-epiphyseal	8	8	11.4	1
Saad et al. [97]	*Medicine (Baltimore)*	Retrospective	66	19.2	All-epiphyseal	18	19	13.3	4
Sasaki et al. [98]	*Orthop J Sports Med*	Retrospective	74	41.6	All-epiphyseal	18	18	12.4	10
				38.1	Trans-epiphyseal	84	84	14.1	75
Seon et al. [99]	*J Korean Med Sci*	Retrospective	58	77.7	Trans-epiphyseal	11	11	14.7	0
Shamrock et al. [100]	*Iowa Orthop J*	Retrospective	60	27.6	Trans-epiphyseal	12	12	12.8	1
Shelbourne et al. [101]	*Am J Sports Med*	Retrospective	61	40.8	Trans-epiphyseal	16	16	14.8	5
Schmale et al. [102]	*Clin Orthop Relat Res*	Retrospective	64	48.0	Trans-epiphyseal	29	29	14.0	23
Streich et al. [103]	*Knee Surg Sports Traumatol Arthrosc*	Retrospective	60	70.0	Trans-epiphyseal	16	16	11.0	6
Wall et al. [104]	*Orthop J Sports Med*	Retrospective	67	43.2	All-epiphyseal	27	27	11.0	4
Willimon et al. [105]	*Am J Sports Med*	Retrospective	66	36.0	All-epiphyseal	21	21	11.8	0
Wren et al. [106]	*Int J Environ Res Public Health*	Retrospective	68	7.8	All-epiphyseal	20	20	11.3	5
Zhang et al. [107]	*Int Orthop*	Retrospective	66	31.6	All-epiphyseal	6	6	12.2	2
				31.6	All-epiphyseal	10	10	12.1	4

### Study characteristics and results of individual studies

Data from 1489 patients (1493 procedures) were collected, of which 32% (490 of 1489 patients) were female. The mean length of the follow-up was 46.6 ± 31.7 months. The mean age of the patients was 12.7 ± 1.1 years. The generalities and demographic of the included studies are presented in Table 1.

### Baseline comparability

Between groups, baseline comparability was evidenced in the length of the follow-up, mean age, female:male ratio, and IKDC and Tegner scores (Table 2).

**Table 2:** Baseline comparability (IKDC)

Endpoint	All-epiphyseal (*N* = 918)	Trans-epiphyseal (*N* = 575)	*P*
Mean follow-up (*months*)	51.4 ± 37.9	40.6 ± 20.8	0.2
Mean age	13.2 ± 1.6	12.1 ± 1.7	0.2
Female (*%*)	40% (369 of 917)	21% (121 of 572)	0.05
IKDC (*mean*)	42.7 ± 3.8	50.4 ± 7.0	0.2
Tegner (*mean*)	5.5 ± 1.9	7.3 ± 0.6	0.3

### Synthesis of results

No difference was found in laxity (Table 3): positive pivot shift (*P* = 0.4), positive Lachman test (*P* = 0.3), and mean arthrometer laxity (*P* = 0.1).

**Table 3:** Results of the outcome: laxity

Endpoint	All-epiphyseal (N = 918)	Trans-epiphyseal (N = 575)	Effect size	*P*
Positive pivot shift test (*%*)	0.1 ± 0.1	0.2 ± 0.4	−0.1	0.4
Arthrometer laxity (*mean*)	2.2 ± 1.6	1.4 ± 0.7	0.8	0.1
• Positive Lachman test (%)	0.2 ± 0.3	0.4 ± 0.5	−0.2	0.3

No difference was found in PROMs (Table 4): IKDC (*P* = 0.3), Lysholm (*P* = 0.4), and Tegner (*P* = 0.7).

**Table 4:** Results of the outcome: PROMs (IKDC)

Endpoint	All-epiphyseal (*N* = 918)	Trans-epiphyseal (*N* = 575)	Effect size	*P*
IKDC (*mean*)	93.6 ± 4.3	84.6 ± 29.0	9.0	0.3
Lysholm (*mean*)	89.4 ± 20.8	95.3 ± 1.6	−5.9	0.4
Tegner (*mean*)	7.2 ± 1.1	7.4 ± 0.8	−0.2	0.7

The trans-epiphyseal technique was associated with a statistically significant rate of patients unable to return to sport (OR 0.1; 95% CI 0.02–0.29; *P* = 0.0001) and with a longer time to return to sport (MD 0.9; 95% CI 0.74–1.05; *P* = 0.01). Though the trans-epiphyseal technique was associated with a lower rate of return to sport, this difference was not statistically significant (*P* = 0.8). No difference was evidenced in the rate of patients who had reduced their league or level of sport activity (*P* = 0.6), and in the rate of patients who had returned to their previous league or level of sports activity (*P* = 0.7). These results are presented in greater detail in Table 5.

**Table 5:** Results of the outcome: return to sport (CI)

Endpoint	All-epiphyseal (*N* = 918)	Trans-epiphyseal (*N* = 575)	95% CI	Effect size	*P*
Return to sport (*n*)	91% (423 of 467)	88% (227 of 258)	0.63–1.43	1.0	0.8
Not able to return to sport (*n*)	1% (3 of 467)	7% (20 of 286)	0.02–0.29	0.1	0.0001
Reduced the level of sport activity or league (*n*)	12% (57 of 467)	14% (36 of 265)	0.56 to 1.38	0.9	0.6
Return to previous level of sport or league (*n*)	82% (383 of 467)	77% (241 of 314)	0.97–1.96	1.4	0.07
Time to return to sport (*months*)	7.7 ± 0.4	8.6 ± 2.3	0.74–1.05	0.9	0.01

No difference was found in the rate of complication (Table 6): re-tear (*P* = 0.8), reoperation (*P* = 0.7), increased laxity (*P* = 0.9), and persistent sensation of instability (*P* = 0.3)

**Table 6:** Results of outcome: complications (CI)

Endpoint	All-epiphyseal (*N* = 918) %	Trans-epiphyseal (*N* = 575) %	Effect size	95% CI	*P*
Re-tear	9 (65 of 685)	10 (42 of 425)	0.63–1.43	0.9560	0.8
Reoperation	11 (46 of 422)	12 (23 of 190)	0.52–1.51	0.888	0.7
Increased laxity	0 (0 of 47)	0 (0 of 36)	0.01–39.65	0.7684	0.9
Persistent sensation of instability	3 (2 of 74)	6 (9 of 160)	0.09–2.21	0.4660	0.3

## Discussion

According to the main findings of the present systematic review, trans-epiphyseal ACL reconstruction was associated with a greater rate of patients unable to return to sport and a longer time to return to sport compared with the all-epiphyseal technique in skeletally immature patients. No differences were found in functional outcomes after surgery. No statistically significant differences were found in complication rate after surgery between the trans-epiphyseal and all-epiphyseal groups.

In the past years, the debate on the appropriate management after ACL rupture in skeletally immature patients has become heated [108]. The main concern regarding the trans-epiphyseal technique was the possible damage to the growth plates [109]. Three different growth disturbances were described [110], namely, the complete arrest of the growth process, depending on the size of the growth plate injury; overgrowth, caused by hypervascularization after the injury; and impaired growth, caused by the tenoepiphysiodesis effect [111]. A recent systematic review of 100 studies analyzed postoperative growth disturbance after ACL reconstruction using trans-epiphyseal techniques [112]. The risk of leg length discrepancy greater than 1 cm was 2.1% and the risk of an angular deformity greater than 5° was 1.3%. To minimize the damage to the physis, tunnels must be as small as possible (< 9 mm), the perichondral ring must be avoided, and the tibial tunnel must be drilled as vertically as possible, preserving the anatomical position of the graft [113]. Pagliazzi et al. [114] conducted a meta-analysis comparing postoperative outcomes after the all-epiphyseal, partial epiphyseal, and trans-epiphyseal techniques. The present systematic review identified no difference in functional outcomes between the three groups. In the all-epiphyseal group, lesser differential laxity than in the other two groups was found. This result was based on only 16 studies, and data on laxity measured by arthrometry were not available. The lower knee laxity did not result in the best functional score nor in the least subjective knee instability, confirming that laxity and instability are different entities. No difference was found in the rate of failure, in accordance with our study.

Petersen et al. [115] analyzed the rate of failure in trans-epiphyseal reconstruction considering the femoral drilling technique. No statistically significant difference was found in re-rupture rate nor in growth disturbance between independent bone tunnels and transtibial tunnels. Instead, the graft choice influenced the rate of failure. The rate of re-rupture was significantly lower using an autologous graft from the extensor apparatus than an autologous graft from the flexor apparatus. This can be explained by the smaller diameter of the gracilis and semitendinosus tendons and their role in limiting anterior tibial translation [116, 117]. Using bone blocks is not recommended in skeletally immature patients because they can bridge the growth plate leading to growth disorder [118]. However, a recent systematic review reported that patients at Tanner stages 3 and 4, who underwent ACLR with a bone-patellar tendon-bone graft, have a 93.8% rate of return to sport [119].

Cordasco et al. [120] conducted a prospective study on children and adolescents, dividing patients into three groups: all-epiphyseal technique in the youngest cohort, trans-epiphyseal and partial trans-epiphyseal technique in young adolescents, and bone-tendon-bone autograft in adolescents at the end of skeletal maturation. Patients of the second group had the highest rate of reoperation and the lowest rate of return to sport. Patients in the first group had a 100% return to sport rate and 92% returned to sport at the same level. This difference may depend on different surgical techniques, as suggested by our results, but also on the high level of competition to which the athletes are exposed during high school. An explanation for the high rate of return to the previous level of sport after all-epiphyseal surgery is given by Ithurburn et al. [121]. They showed that, when returning to sport, young athletes after all-epiphyseal ACLR demonstrated higher quadriceps strength symmetry and knee-related function than adolescents after trans-epiphyseal ACLR. Kay et al. [122], in a meta-analysis, found a 92% return to sport rate and that 76% of the patients returned to sport at the previous level. No difference was found between the trans-epiphyseal group and the all-epiphyseal group, but only four studies on the all-epiphyseal group were examined. In an 8-year follow-up study, early return to sport is an important risk factor for a second ACL injury [11]. Rehabilitation after ACLR requires more time for children than for adults [123]. Return to sport should be postponed 9 months after surgery, and 12 months for pivoting sports [113, 123].

The present study has some limitations. Firstly, the retrospective nature of the included studies and the methodological quality assessment highlighted the fair quality of the included studies. Several sources of heterogeneity must be highlighted. The surgical protocols and the absence of a standardized postoperative rehabilitation program represent important sources of bias. Indeed, the evaluation of the return to sport has no accepted criteria, and the choice to abandon sports activity can be independent of knee condition. There was no homogeneity in sex between the two cohorts of patients. There was a statistically significant difference in the number of female patients who underwent trans-epiphyseal and all–epiphyseal ACL reconstruction. A previous meta-analysis found similar results [124]. The authors hypothesized that the faster maturation of the female skeleton could explain this difference. Some authors [59, 61, 72, 74] used allografts for the reconstruction; whether ACL reconstruction using allografts is associated with a difference in the outcome is controversial. Allografts avoid the harvesting site, which could promote faster recovery and shorter operation time. On the contrary, allografts have a higher risk of rejection and infection. The authors used heterogeneous types of autografts. Among them, hamstring [57, 58, 62, 64–68, 75–77, 79, 82–85, 89, 92–95, 97, 99, 100, 104, 106, 107], patellar [60, 63, 87, 88, 96, 101, 106] and iliotibial band [80, 90, 91, 105, 106] autografts were most commonly used. Given the lack of quantitative data and limited information, the types of autografts used could not be analyzed separately. Several all-epiphyseal ACL reconstruction techniques have been described, including the Anderson, Ganley–Lawrence, and Cordasco–Green [31, 43–45]. Given the lack of quantitative data and missing information on the surgical approach, it was not possible to analyze the different all- and trans-epiphyseal techniques. Given the lack of information regarding the reasons for reoperations, additional analyses on this endpoint were not possible to develop. Future investigations are required to compare all- versus trans-epiphyseal ACL reconstruction, validating the results of the present study in a clinical setting.

## Conclusion

Trans-epiphyseal ACL reconstruction was associated with a greater rate of patients unable to return to sport and with a longer time to return to sport compared with the all-epiphyseal technique in skeletally immature patients.

## Data Availability

The datasets generated during and/or analyzed during the current study are available throughout the manuscript.

## References

[1] Gianotti SM, Marshall SW, Hume PA, Bunt L (2009). Incidence of anterior cruciate ligament injury and other knee ligament injuries: a national population-based study. J Sci Med Sport.

[2] Maffulli N, Loppini M, King JB (2013). Anterior cruciate ligament tears: what we already know. Knee Surg Sports Traumatol Arthrosc.

[3] Clayton RA, Court-Brown CM (2008). The epidemiology of musculoskeletal tendinous and ligamentous injuries. Injury.

[4] Meighan AA, Keating JF, Will E (2003). Outcome after reconstruction of the anterior cruciate ligament in athletic patients a comparison of early versus delayed surgery. J Bone Joint Surg Br.

[5] Frank CB, Jackson DW (1997). The science of reconstruction of the anterior cruciate ligament. J Bone Joint Surg Am.

[6] Arendt EA, Agel J, Dick R (1999). Anterior cruciate ligament injury patterns among collegiate men and women. J Athl Train.

[7] Agel J, Arendt EA, Bershadsky B (2005). Anterior cruciate ligament injury in national collegiate athletic association basketball and soccer: a 13-year review. Am J Sports Med.

[8] Turati M, Rigamonti L, Giulivi A, Gaddi D, Accadbled F, Zanchi N, Bremond N, Catalano M, Gorla M, Omeljaniuk RJ, Zatti G, Piatti M, Bigoni M (2021). Management of anterior cruciate ligament tears in Tanner stage 1 and 2 children: a narrative review and treatment algorithm guided by ACL tear location. J Sports Med Phys Fitness.

[9] Dingel A, Aoyama J, Ganley T, Shea K (2019). Pediatric ACL tears: natural history. J Pediatr Orthop.

[10] Dodwell ER, Lamont LE, Green DW, Pan TJ, Marx RG, Lyman S (2014). 20 years of pediatric anterior cruciate ligament reconstruction in New York State. Am J Sports Med.

[11] Dekker TJ, Godin JA, Dale KM, Garrett WE, Taylor DC, Riboh JC (2017). Return to sport after pediatric anterior cruciate ligament reconstruction and its effect on subsequent anterior cruciate ligament injury. J Bone Joint Surg Am.

[12] Shaw L, Finch CF (2017). Trends in pediatric and adolescent anterior cruciate ligament injuries in Victoria, Australia 2005–2015. Int J Environ Res Public Health.

[13] Prodromos CC, Han Y, Rogowski J, Joyce B, Shi K (2007). A meta-analysis of the incidence of anterior cruciate ligament tears as a function of gender, sport, and a knee injury-reduction regimen. Arthroscopy.

[14] Van de Velde SK, Gill TJ, Li G (2009). Evaluation of kinematics of anterior cruciate ligament-deficient knees with use of advanced imaging techniques, three-dimensional modeling techniques, and robotics. J Bone Joint Surg Am.

[15] Chaudhari AM, Briant PL, Bevill SL, Koo S, Andriacchi TP (2008). Knee kinematics, cartilage morphology, and osteoarthritis after ACL injury. Med Sci Sports Exerc.

[16] Ferber R, Osternig LR, Woollacott MH, Wasielewski NJ, Lee JH (2002). Gait mechanics in chronic ACL deficiency and subsequent repair. Clin Biomech (Bristol, Avon).

[17] Andersson C, Odensten M, Gillquist J (1991). Knee function after surgical or nonsurgical treatment of acute rupture of the anterior cruciate ligament: a randomized study with a long-term follow-up period. Clin Orthop Relat Res.

[18] Fink C, Hoser C, Benedetto KP (1993). Sports capacity after rupture of the anterior cruciate ligament–surgical versus non-surgical therapy. Aktuelle Traumatol.

[19] Fink C, Hoser C, Benedetto KP (1994). Development of arthrosis after rupture of the anterior cruciate ligament a comparison of surgical and conservative therapy. Unfallchirurg..

[20] Frobell RB, Lohmander LS, Roos EM (2007). The challenge of recruiting patients with anterior cruciate ligament injury of the knee into a randomized clinical trial comparing surgical and non-surgical treatment. Contemp Clin Trials.

[21] Hinterwimmer S, Engelschalk M, Sauerland S, Eitel F, Mutschler W (2003). Operative or conservative treatment of anterior cruciate ligament rupture: a systematic review of the literature. Unfallchirurg.

[22] Jerosch J, Schaffer C, Prymka M (1998). Proprioceptive abilities of surgically and conservatively treated knee joints with injuries of the cruciate ligament. Unfallchirurg.

[23] Meunier A, Odensten M, Good L (2007). Long-term results after primary repair or non-surgical treatment of anterior cruciate ligament rupture: a randomized study with a 15-year follow-up. Scand J Med Sci Sports.

[24] Odensten M, Hamberg P, Nordin M, Lysholm J, Gillquist J (1985). Surgical or conservative treatment of the acutely torn anterior cruciate ligament. a randomized study with short-term follow-up observations. Clin Orthop Relat Res.

[25] Scavenius M, Bak K, Hansen S, Norring K, Jensen KH, Jorgensen U (1999). Isolated total ruptures of the anterior cruciate ligament–a clinical study with long-term follow-up of 7 years. Scand J Med Sci Sports.

[26] Seitz H, Chrysopoulos A, Egkher E, Mousavi M (1994). Long-term results of replacement of the anterior cruciate ligament in comparison with conservative therapy. Chirurg.

[27] Zysk SP, Refior HJ (2000). Operative or conservative treatment of the acutely torn anterior cruciate ligament in middle-aged patients a follow-up study of 133 patients between the ages of 40 and 59 years. Arch Orthop Trauma Surg.

[28] van Meer BL, Oei EH, Meuffels DE, van Arkel ER, Verhaar JA, Bierma-Zeinstra SM, Reijman M (2016). Degenerative changes in the knee 2 years after anterior cruciate ligament rupture and related risk factors: a prospective observational follow-up study. Am J Sports Med.

[29] Bottoni CR, Liddell TR, Trainor TJ, Freccero DM, Lindell KK (2008). Postoperative range of motion following anterior cruciate ligament reconstruction using autograft hamstrings: a prospective, randomized clinical trial of early versus delayed reconstructions. Am J Sports Med.

[30] Kostogiannis I, Ageberg E, Neuman P, Dahlberg L, Friden T, Roos H (2007). Activity level and subjective knee function 15 years after anterior cruciate ligament injury: a prospective, longitudinal study of nonreconstructed patients. Am J Sports Med.

[31] Perkins CA, Willimon SC (2020). Pediatric anterior cruciate ligament reconstruction. Orthop Clin North Am.

[32] Kercher J, Xerogeanes J, Tannenbaum A, Al-Hakim R, Black JC, Zhao J (2009). Anterior cruciate ligament reconstruction in the skeletally immature: an anatomical study utilizing 3-dimensional magnetic resonance imaging reconstructions. J Pediatr Orthop.

[33] Willson RG, Kostyun RO, Milewski MD, Nissen CW (2018). Anterior cruciate ligament reconstruction in skeletally immature patients: early results using a hybrid physeal-sparing technique. Orthop J Sports Med.

[34] Migliorini F, Maffulli N, Bell A, Betsch M (2022). Outcomes, return to sport, and failures of mpfl reconstruction using autografts in children and adolescents with recurrent patellofemoral instability: a systematic review. Children.

[35] Migliorini F, La Padula G, Oliva F, Torsiello E, Hildebrand F, Maffulli N (2022). Operative management of avascular necrosis of the femoral head in skeletally immature patients: a systematic review. Life.

[36] Migliorini F, Rath B, Tingart M, Meisen N, Eschweiler J (2019). Surgical management for recurrent patellar dislocations in skeletally immature patients. Eur J Orthop Surg Traumatol.

[37] Kaeding CC, Flanigan D, Donaldson C (2010). Surgical techniques and outcomes after anterior cruciate ligament reconstruction in preadolescent patients. Arthroscopy.

[38] Kocher MS, Saxon HS, Hovis WD, Hawkins RJ (2002). Management and complications of anterior cruciate ligament injuries in skeletally immature patients: survey of the herodicus society and The ACL Study Group. J Pediatr Orthop.

[39] Koman JD, Sanders JO (1999). Valgus deformity after reconstruction of the anterior cruciate ligament in a skeletally immature patient a case report. J Bone Joint Surg Am.

[40] Migliorini F, Eschweiler J, Mansy YE, Quack V, Tingart M, Driessen A (2020). Quadriceps tendon autograft for primary ACL reconstruction: a Bayesian network meta-analysis. Eur J Orthop Surg Traumatol.

[41] Migliorini F, Eschweiler J, Mansy YE, Quack V, Tingart M, Driessen A (2021). Correction to: Quadriceps tendon autograft for primary ACL reconstruction: a Bayesian network meta-analysis. Eur J Orthop Surg Traumatol.

[42] Migliorini F, Eschweiler J, Tingart M, Niewiera M, Rath B (2020). Bone-patellar tendon-bone versus four strands hamstring grafts for anterior cruciate ligament reconstruction. Muscle Ligament Tendon J.

[43] Anderson AF (2004). Transepiphyseal replacement of the anterior cruciate ligament using quadruple hamstring grafts in skeletally immature patients. J Bone Joint Surg Am.

[44] Lawrence JT, Bowers AL, Belding J, Cody SR, Ganley TJ (2010). All-epiphyseal anterior cruciate ligament reconstruction in skeletally immature patients. Clin Orthop Relat Res.

[45] McCarthy MM, Graziano J, Green DW, Cordasco FA (2012). All-epiphyseal, all-inside anterior cruciate ligament reconstruction technique for skeletally immature patients. Arthrosc Tech.

[46] Connaughton AJ, Geeslin AG, Uggen CW (2017). All-inside ACL reconstruction: how does it compare to standard ACL reconstruction techniques?. J Orthop.

[47] Howick JCI, Glasziou P, Greenhalgh T, Heneghan C, Liberati A, Moschetti I, Phillips B, Thornton H, Goddard O, Hodgkinson M (2011). The 2011 Oxford CEBM levels of evidence.

[48] Page MJ, McKenzie JE, Bossuyt PM, Boutron I, Hoffmann TC, Mulrow CD, Shamseer L, Tetzlaff JM, Akl EA, Brennan SE, Chou R, Glanville J, Grimshaw JM, Hrobjartsson A, Lalu MM, Li T, Loder EW, Mayo-Wilson E, McDonald S, McGuinness LA, Stewart LA, Thomas J, Tricco AC, Welch VA, Whiting P, Moher D (2021). The PRISMA 2020 statement: an updated guideline for reporting systematic reviews. BMJ.

[49] Briggs KK, Lysholm J, Tegner Y, Rodkey WG, Kocher MS, Steadman JR (2009). The reliability, validity, and responsiveness of the Lysholm score and Tegner activity scale for anterior cruciate ligament injuries of the knee: 25 years later. Am J Sports Med.

[50] Lysholm J, Gillquist J (1982). Evaluation of knee ligament surgery results with special emphasis on use of a scoring scale. Am J Sports Med.

[51] Higgins LD, Taylor MK, Park D, Ghodadra N, Marchant M, Pietrobon R, Cook Chad (2007). Reliability and validity of the international knee documentation committee (IKDC) subjective knee form. Joint Bone Spine.

[52] Mostafaee N, Negahban H, Shaterzadeh Yazdi MJ, Goharpey S, Mehravar M, Pirayeh N (2020). Responsiveness of a Persian version of knee injury and osteoarthritis outcome score and Tegner activity scale in athletes with anterior cruciate ligament reconstruction following physiotherapy treatment. Physiother Theory Pract.

[53] Jones KJ, Kelley BV, Arshi A, McAllister DR, Fabricant PD (2019). Comparative effectiveness of cartilage repair with respect to the minimal clinically important difference. Am J Sports Med.

[54] Agarwalla A, Liu JN, Garcia GH, Gowd AK, Puzzitiello RN, Yanke AB, Cole BJ (2021). Return to sport following isolated lateral opening wedge distal femoral osteotomy. Cartilage.

[55] Coleman BD, Khan KM, Maffulli N, Cook JL, Wark JD (2000). Studies of surgical outcome after patellar tendinopathy: clinical significance of methodological deficiencies and guidelines for future studies Victorian Institute of Sport Tendon Study Group. Scand J Med Sci Sports.

[56] Higgins JPT TJ, Chandler J, Cumpston M, Li T, Page MJ, Welch VA . Cochrane Handbook for Systematic Reviews of Interventions version 6.2. Cochrane 2021. www.training.cochrane.org/handbook. Accessed on February 2022.

[57] Aichroth PM, Patel DV, Zorrilla P (2002). The natural history and treatment of rupture of the anterior cruciate ligament in children and adolescents a prospective review. J Bone Joint Surg Br.

[58] Akinleye SD, Sewick A, Wells L (2013). All-epiphyseal acl reconstruction: a three-year follow-up. Int J Sports Phys Ther.

[59] Andrews M, Noyes FR, Barber-Westin SD (1994). Anterior cruciate ligament allograft reconstruction in the skeletally immature athlete. Am J Sports Med.

[60] Arbes S, Resinger C, Vecsei V, Nau T (2007). The functional outcome of total tears of the anterior cruciate ligament (ACL) in the skeletally immature patient. Int Orthop.

[61] Aronowitz ER, Ganley TJ, Goode JR, Gregg JR, Meyer JS (2000). Anterior cruciate ligament reconstruction in adolescents with open physes. Am J Sports Med.

[62] Asai K, Nakase J, Shimozaki K, Yoshimizu R, Kimura M, Tsuchiya H (2021). Skeletally immature patient showed lower graft maturity than skeletally mature patient after ACL reconstruction with a rounded rectangular femoral tunnel. Sci Rep.

[63] Bonnard C, Fournier J, Babusiaux D, Planchenault M, Bergerault F, de Courtivron B (2011). Physeal-sparing reconstruction of anterior cruciate ligament tears in children: results of 57 cases using patellar tendon. J Bone Joint Surg Br.

[64] Calvo R, Figueroa D, Gili F, Vaisman A, Mococain P, Espinosa M, Leon A, Arellano S (2015). Transphyseal anterior cruciate ligament reconstruction in patients with open physes: 10-year follow-up study. Am J Sports Med.

[65] Cassard X, Cavaignac E, Maubisson L, Bowen M (2014). Anterior cruciate ligament reconstruction in children with a quadrupled semitendinosus graft: preliminary results with minimum 2 years of follow-up. J Pediatr Orthop.

[66] Cohen M, Ferretti M, Quarteiro M, Marcondes FB, de Hollanda JP, Amaro JT, Abdalla RJ (2009). Transphyseal anterior cruciate ligament reconstruction in patients with open physes. Arthroscopy.

[67] Courvoisier A, Grimaldi M, Plaweski S (2011). Good surgical outcome of transphyseal ACL reconstruction in skeletally immature patients using four-strand hamstring graft. Knee Surg Sports Traumatol Arthrosc.

[68] Cordasco FA, Mayer SW, Green DW (2017). All-inside, all-epiphyseal anterior cruciate ligament reconstruction in skeletally immature athletes: return to sport, incidence of second surgery, and 2-year clinical outcomes. Am J Sports Med.

[69] Cruz AI, Fabricant PD, McGraw M, Rozell JC, Ganley TJ, Wells L (2017). All-epiphyseal ACL reconstruction in children: review of safety and early complications. J Pediatr Orthop.

[70] Demange MK, Camanho GL (2014). Nonanatomic anterior cruciate ligament reconstruction with double-stranded semitendinosus grafts in children with open physes: minimum 15-year follow-up. Am J Sports Med.

[71] Foissey C, Thaunat M, Caron E, Haidar I, Vieira TD, Gomes L, Freychet B, Sonnery-Cottet B, Fayard JM (2022). Combining anterior cruciate ligament reconstruction with lateral extra-articular procedures in skeletally immature patients is safe and associated with a low failure rate. Arthrosc Sports Med Rehabil.

[72] Fuchs R, Wheatley W, Uribe JW, Hechtman KS, Zvijac JE, Schurhoff MR (2002). Intra-articular anterior cruciate ligament reconstruction using patellar tendon allograft in the skeletally immature patient. Arthroscopy.

[73] Gebhard F, Ellermann A, Hoffmann F, Jaeger JH, Friederich NF (2006). Multicenter-study of operative treatment of intraligamentous tears of the anterior cruciate ligament in children and adolescents: comparison of four different techniques. Knee Surg Sports Traumatol Arthrosc.

[74] Goddard M, Bowman N, Salmon LJ, Waller A, Roe JP, Pinczewski LA (2013). Endoscopic anterior cruciate ligament reconstruction in children using living donor hamstring tendon allografts. Am J Sports Med.

[75] Greenberg EM, Greenberg ET, Ganley TJ, Lawrence JT (2014). Strength and functional performance recovery after anterior cruciate ligament reconstruction in preadolescent athletes. Sports Health.

[76] Guzzanti V, Falciglia F, Stanitski CL (2003). Physeal-sparing intraarticular anterior cruciate ligament reconstruction in preadolescents. Am J Sports Med.

[77] Hoshikawa A, Hiraoka H, Monobe Y, Shiraki K, Sasaki Y, Nakamura H, Saita K, Sakai H (2020). Midterm clinical results after all-epiphyseal double-bundle reconstruction of the anterior cruciate ligament in children with open physes. Orthop J Sports Med.

[78] Hui C, Roe J, Ferguson D, Waller A, Salmon L, Pinczewski L (2012). Outcome of anatomic transphyseal anterior cruciate ligament reconstruction in Tanner stage 1 and 2 patients with open physes. Am J Sports Med.

[79] Koch PP, Fucentese SF, Blatter SC (2016). Complications after epiphyseal reconstruction of the anterior cruciate ligament in prepubescent children. Knee Surg Sports Traumatol Arthrosc.

[80] Kocher MS, Garg S, Micheli LJ (2006). Physeal sparing reconstruction of the anterior cruciate ligament in skeletally immature prepubescent children and adolescents surgical technique. J Bone Joint Surg Am.

[81] Kohl S, Stutz C, Decker S, Ziebarth K, Slongo T, Ahmad SS, Kohlhof H, Eggli S, Zumstein M, Evangelopoulos DS (2014). Mid-term results of transphyseal anterior cruciate ligament reconstruction in children and adolescents. Knee.

[82] Kumar S, Ahearne D, Hunt DM (2013). Transphyseal anterior cruciate ligament reconstruction in the skeletally immature: follow-up to a minimum of sixteen years of age. J Bone Joint Surg Am.

[83] Lanzetti RM, Pace V, Ciompi A, Perugia D, Spoliti M, Falez F, Auro C (2020). Over the top anterior cruciate ligament reconstruction in patients with open physes: a long-term follow-up study. Int Orthop.

[84] Lemaitre G, Salle de Chou E, Pineau V, Rochcongar G, Delforge S, Bronfen C, Haumont T, Hulet C (2014). ACL reconstruction in children: a transphyseal technique. Orthop Traumatol Surg Res.

[85] Liddle AD, Imbuldeniya AM, Hunt DM (2008). Transphyseal reconstruction of the anterior cruciate ligament in prepubescent children. J Bone Joint Surg Br.

[86] Mauch C, Arnold MP, Wirries A, Mayer RR, Friederich NF, Hirschmann MT (2011). Anterior cruciate ligament reconstruction using quadriceps tendon autograft for adolescents with open physes- a technical note. Sports Med Arthrosc Rehabil Ther Technol.

[87] McCarroll JR, Rettig AC, Shelbourne KD (1988). Anterior cruciate ligament injuries in the young athlete with open physes. Am J Sports Med.

[88] McCarroll JR, Shelbourne KD, Porter DA, Rettig AC, Murray S (1994). Patellar tendon graft reconstruction for midsubstance anterior cruciate ligament rupture in junior high school athletes an algorithm for management. Am J Sports Med.

[89] McIntosh AL, Dahm DL, Stuart MJ (2006). Anterior cruciate ligament reconstruction in the skeletally immature patient. Arthroscopy.

[90] Micheli LJ, Rask B, Gerberg L (1999). Anterior cruciate ligament reconstruction in patients who are prepubescent. Clin Orthop Relat Res.

[91] Nakhostine M, Bollen SR, Cross MJ (1995). Reconstruction of mid-substance anterior cruciate rupture in adolescents with open physes. J Pediatr Orthop.

[92] Nikolaou P, Kalliakmanis A, Bousgas D, Zourntos S (2011). Intraarticular stabilization following anterior cruciate ligament injury in children and adolescents. Knee Surg Sports Traumatol Arthrosc.

[93] Perelli S, Costa GG, Terron VM, Formagnana M, Bait C, Espregueira-Mendes J, Monllau JC (2022). Combined anterior cruciate ligament reconstruction and modified lemaire lateral extra-articular tenodesis better restores knee stability and reduces failure rates than isolated anterior cruciate ligament reconstruction in skeletally immature patients. Am J Sports Med.

[94] Pennock AT, Chambers HG, Turk RD, Parvanta KM, Dennis MM, Edmonds EW (2018). Use of a modified all-epiphyseal technique for anterior cruciate ligament reconstruction in the skeletally immature patient. Orthop J Sports Med.

[95] Redler LH, Brafman RT, Trentacosta N, Ahmad CS (2012). Anterior cruciate ligament reconstruction in skeletally immature patients with transphyseal tunnels. Arthroscopy.

[96] Robert H, Bonnard C (1999). The possibilities of using the patellar tendon in the treatment of anterior cruciate ligament tears in children. Arthroscopy.

[97] Saad L, Grimard G, Nault ML (2021). Complication rates following all-epiphyseal ACL reconstructions in skeletally immature patients: a retrospective case series study. Medicine (Baltimore).

[98] Sasaki S, Sasaki E, Kimura Y, Yamamoto Y, Tsuda E, Ishibashi Y (2021). Clinical outcomes and postoperative complications after all-epiphyseal double-bundle ACL reconstruction for skeletally immature patients. Orthop J Sports Med.

[99] Seon JK, Song EK, Yoon TR, Park SJ (2005). Transphyseal reconstruction of the anterior cruciate ligament using hamstring autograft in skeletally immature adolescents. J Korean Med Sci.

[100] Shamrock AG, Duchman KR, Cates WT, Cates RA, Khazi ZM, Westermann RW, Bollier MJ, Wolf BR (2022). Outcomes following primary anterior cruciate ligament reconstruction using a partial transphyseal (over-the-top) technique in skeletally immature patients. Iowa Orthop J.

[101] Shelbourne KD, Gray T, Wiley BV (2004). Results of transphyseal anterior cruciate ligament reconstruction using patellar tendon autograft in Tanner stage 3 or 4 adolescents with clearly open growth plates. Am J Sports Med.

[102] Schmale GA, Kweon C, Larson RV, Bompadre V (2014). High satisfaction yet decreased activity 4 years after transphyseal ACL reconstruction. Clin Orthop Relat Res.

[103] Streich NA, Barie A, Gotterbarm T, Keil M, Schmitt H (2010). Transphyseal reconstruction of the anterior cruciate ligament in prepubescent athletes. Knee Surg Sports Traumatol Arthrosc.

[104] Wall EJ, Ghattas PJ, Eismann EA, Myer GD, Carr P (2017). Outcomes and complications after all-epiphyseal anterior cruciate ligament reconstruction in skeletally immature patients. Orthop J Sports Med.

[105] Willimon SC, Jones CR, Herzog MM, May KH, Leake MJ, Busch MT (2015). Micheli anterior cruciate ligament reconstruction in skeletally immature youths: a retrospective case series with a mean 3-year follow-up. Am J Sports Med.

[106] Wren TL, Beltran V, Katzel MJ, Conrad-Forrest AS, VandenBerg CD (2021). Iliotibial band autograft provides the fastest recovery of knee extensor mechanism function in pediatric anterior cruciate ligament reconstruction. Int J Environ Res Public Health.

[107] Zhang L, Liang Q, Zhao Z, Zhang L, Kang X, Tian B, Ren B, Zhang X, Gao Z, Wang Y, Zheng J (2023). Robot-assisted all-epiphyseal anterior cruciate ligament reconstruction in skeletally immature patients: a retrospective study. Int Orthop.

[108] Seil R, Chotel F, Robert H (2019). Collaborative efforts are needed to gain new knowledge on pediatric and adolescent anterior cruciate ligament (ACL) injuries. Orthop Traumatol Surg Res.

[109] Rohde MS, Cinque ME, LaPrade CM, Ganley TJ, Shea KG (2022). The spectrum of anterior cruciate ligament reconstruction options for the pediatric and adolescent patient: a narrative review. J Athl Train.

[110] Rybak LP, Whitworth C, Scott V, Weberg AD, Bhardwaj B (1991). Rat as a potential model for hearing loss in biotinidase deficiency. Ann Otol Rhinol Laryngol.

[111] Chotel F, Henry J, Seil R, Chouteau J, Moyen B, Berard J (2010). Growth disturbances without growth arrest after ACL reconstruction in children. Knee Surg Sports Traumatol Arthrosc.

[112] Fury MS, Paschos NK, Fabricant PD, Anderson CN, Busch MT, Kocher MS (2022). Assessment of skeletal maturity and postoperative growth disturbance after anterior cruciate ligament reconstruction in skeletally immature patients: a systematic review. Am J Sports Med..

[113] Ardern CL, Ekas G, Grindem H, Moksnes H, Anderson A, Chotel F, Cohen M, Forssblad M, Ganley TJ, Feller JA, Karlsson J, Kocher MS, LaPrade RF, McNamee M, Mandelbaum B, Micheli L, Mohtadi N, Reider B, Roe J, Seil R, Siebold R, Silvers-Granelli HJ, Soligard T, Witvrouw E, Engebretsen L (2018). 2018 International Olympic Committee consensus statement on prevention, diagnosis and management of paediatric anterior cruciate ligament (ACL) injuries. Knee Surg Sports Traumatol Arthrosc.

[114] Pagliazzi G, Cuzzolin M, Pacchiarini L, Delcogliano M, Filardo G, Candrian C (2023). Physeal-sparing ACL reconstruction provides better knee laxity restoration but similar clinical outcomes to partial transphyseal and complete transphyseal approaches in the pediatric population: a systematic review and meta-analysis. Knee Surg Sports Traumatol Arthrosc.

[115] Petersen W, Bierke S, Stohr A, Stoffels T, Haner M (2023). A systematic review of transphyseal ACL reconstruction in children and adolescents: comparing the transtibial and independent femoral tunnel drilling techniques. J Exp Orthop.

[116] Conte EJ, Hyatt AE, Gatt CJ, Dhawan A (2014). Hamstring autograft size can be predicted and is a potential risk factor for anterior cruciate ligament reconstruction failure. Arthroscopy.

[117] Haddara R, Harandi VJ, Lee PVS (2020). Anterior cruciate ligament agonist and antagonist muscle force differences between males and females during perturbed walking. J Biomech.

[118] Seil R, Weitz FK, Pape D (2015). Surgical-experimental principles of anterior cruciate ligament (ACL) reconstruction with open growth plates. J Exp Orthop.

[119] Turati M, Caliandro M, Gaddi D, Piatti M, Rigamonti L, Zanchi N, Di Benedetto P, Boerci L, Catalano M, Zatti G, Ollivier M, Bigoni M (2022). Clinical outcomes and complications after anterior cruciate ligament reconstruction with bone-patellar tendon-bone in patient Tanner 3 and 4: a systematic review. Eur J Orthop Surg Traumatol.

[120] Cordasco FA, Black SR, Price M, Wixted C, Heller M, Asaro LA, Nguyen J, Green DW (2019). Return to sport and reoperation rates in patients under the age of 20 after primary anterior cruciate ligament reconstruction: risk profile comparing 3 patient groups predicated upon skeletal age. Am J Sports Med.

[121] Ithurburn MP, Paljieg A, Thomas S, Hewett TE, Paterno MV, Schmitt LC (2019). Strength and function across maturational levels in young athletes at the time of return to sport after ACL reconstruction. Sports Health.

[122] Kay J, Memon M, Marx RG, Peterson D, Simunovic N, Ayeni OR (2018). Over 90 % of children and adolescents return to sport after anterior cruciate ligament reconstruction: a systematic review and meta-analysis. Knee Surg Sports Traumatol Arthrosc.

[123] Hansson F, Mostrom EB, Forssblad M, Stalman A, Janarv PM (2022). Long-term evaluation of pediatric ACL reconstruction: high risk of further surgery but a restrictive postoperative management was related to a lower revision rate. Arch Orthop Trauma Surg.

[124] Pierce TP, Issa K, Festa A, Scillia AJ, McInerney VK (2017). Pediatric anterior cruciate ligament reconstruction: a systematic review of transphyseal versus physeal-sparing techniques. Am J Sports Med.

